# The role of the NLRP3 inflammasome in chronic inflammation in asthma and chronic obstructive pulmonary disease

**DOI:** 10.1002/iid3.750

**Published:** 2022-11-25

**Authors:** Yaxin Wu, Xin Di, Min Zhao, Haoran Li, Li Bai, Ke Wang

**Affiliations:** ^1^ Department of Respiratory and Critical Care Medicine The Second Hospital of Jilin University Changchun China

**Keywords:** asthma, chronic inflammation, COPD, inflammasome, NLRP3

## Abstract

Asthma and chronic obstructive pulmonary disease (COPD) are lung diseases characterized by airflow limitation and chronic inflammation. More and more studies have shown that the occurrence and development of asthma and COPD are related to abnormal immune responses caused by dysregulation of many genetic and environmental factors. The exact pathogenesis of the disease is still unclear. A large number of studies have shown that the NLRP3 inflammasome is involved in the process of chronic airway inflammation in asthma and COPD. Here, we summarize recent advances in the mechanism of NLRP3 inflammasome activation and regulation and its role in the pathogenesis of inflammatory lung diseases such as asthma and COPD. Meanwhile we propose possible therapeutic targets in asthma and COPD.

## Graphical abstract

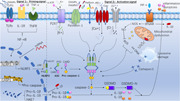

## 1 INTRODUCTION

Asthma and chronic obstructive pulmonary disease (COPD) are the most common chronic noncommunicable diseases. A meta‐analysis of 27 studies showed that the global prevalence rate of asthma was 6.2% (95% confidence interval [CI]: 5.0%–7.4%) and that of COPD was 4.9% (95% CI:4.3%–5.5%).^1^ Some studies have found that the prevalence rate of children and adolescents in asthma is increasing year by year, which may be related to maternal smoking and early antibiotic exposure.^2^ Although asthma is more common, COPD is associated with higher mortality. It is estimated that 328 million people worldwide suffer from COPD.^3^ In 2020, COPD has become the third leading cause of death in the world, second only to cardiovascular disease and tumors.^4^ Asthma and COPD bring huge social, economic and medical burdens. Some studies predict that uncontrollable asthma among adolescents and adults in the United States will cause an economic burden of $963.5 billion in the next 20 years, with per capita costs ranging from $2209 to $6132.^5^ Direct medical expenses for COPD patients are estimated at $800.9 billion in the next 20 years.^6^ According to a survey in Europe, the annual direct cost of COPD patients ranges from 10701 euros to 1963 euros.^7^ A study from the Asia‐Pacific region also shows that the annual cost of COPD patients ranges from $4398 to $23049 per capita.^8^ In low‐and middle‐income countries that lack proper treatment, asthma and COPD put serious pressure on patients, their families and society. Despite the significant improvement in treatment and medical level in recent decades, the pathogenesis of asthma and COPD is still a research hotspot.

Studies have found that NLRP3 inflammasomes are closely related to a variety of lung diseases, such as asthma, COPD, pulmonary fibrosis, acute lung injury and lung cancer. A growing number of studies have shown that NLRP3 inflammasomes are involved in the process of chronic airway inflammation in asthma and COPD.^9^, ^10^ In diseased lungs, we believe that inflammasomes are inappropriately activated, resulting in chronic inflammation.^11^ However, the detailed role of NLRP3 inflammasome in asthma and COPD remains controversial. The purpose of this review is to collate recent advances in the activation and regulation of the NLRP3 inflammasome and the evidence for the relationship between the NLRP3 inflammasome and lung diseases, especially literature reports related to asthma and COPD. We discuss the complex relationship between NLRP3 inflammasomes and inflammatory responses found in animal models, asthma and COPD patients, to prove that inflammasome plays a key role in asthma and COPD inflammation. At the same time, a potential therapeutic target for inhibiting the activation of NLRP3 inflammasome is proposed, which provides a new direction for the treatment of asthma and COPD.

## 2 THE NLRP3 INFLAMMASOME

The inflammasome is a multi‐protein complex present in the cytoplasm that can be activated by various pathogen‐associated molecular patterns (PAMPs) such as lipopolysaccharide (LPS), bacterial and viral RNA, and damage‐associated molecular patterns (DAMPs) such as reactive oxygen species (ROS), adenosine triphosphate (ATP), and mitochondrial DNA, as well as homeostasis‐altering molecular patterns (HAMPs) that detect changes in cellular homeostasis and trigger an inflammatory response in the host.^12^, ^13^, ^14^ The inflammasome that NOD‐like receptor pyrin domain‐related protein 3 (NLRP3) participates in is called the NLRP3 inflammasome.

### 2.1 Composition of the NLRP3 inflammasome

**Figure 1:**
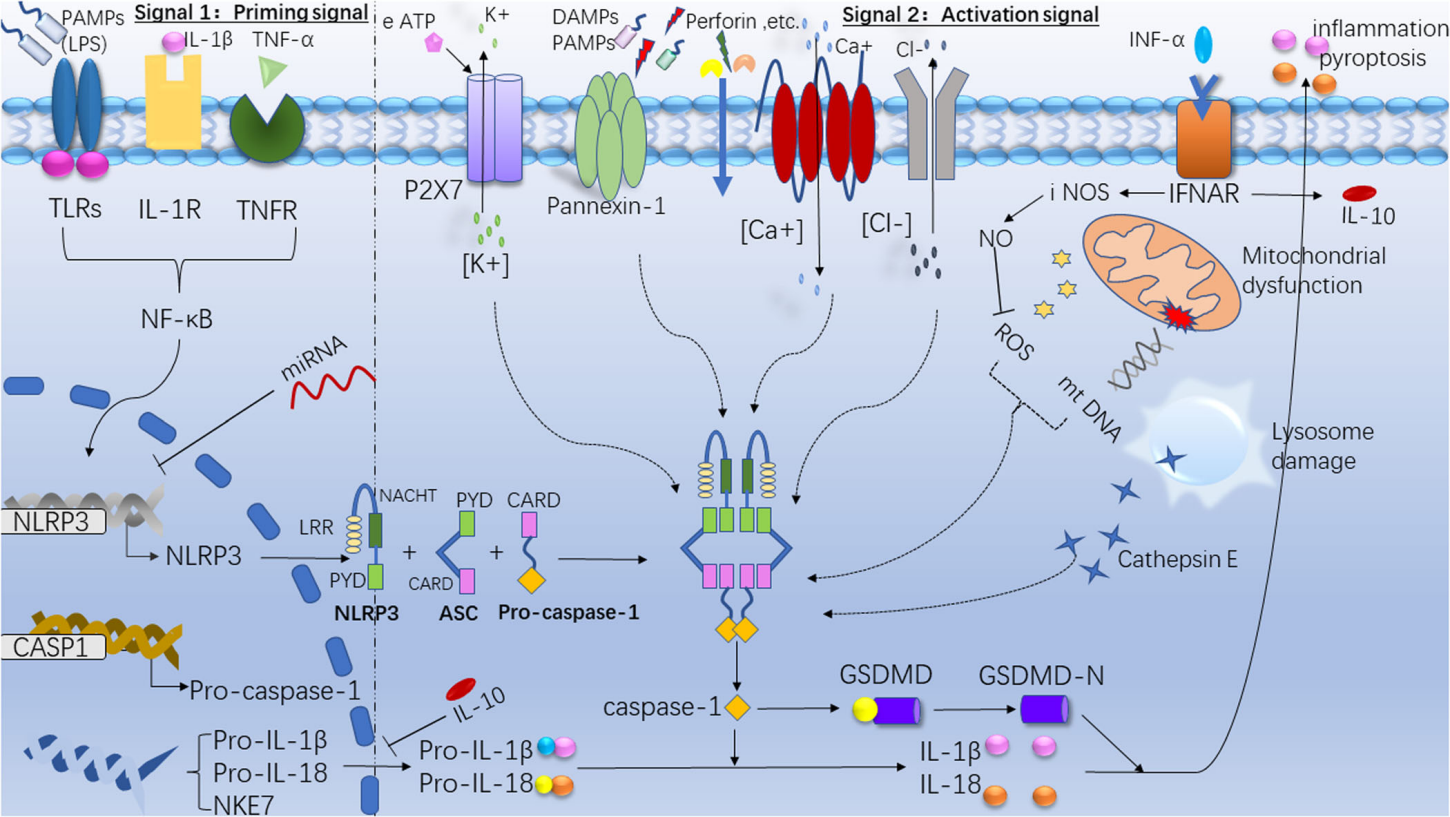
The composition, activation, and regulation of the NLRP3 inflammasome. The signal 1 (priming signal; left) is provided by the activation of cytokines (IL‐1β; tumor necrosis factor [TNF]‐α) or pathogen‐associated molecular patterns (PAMPs), leading to the transcriptional upregulation of NLRP3 inflammasome components (NLRP3 receptor protein; ASC; pro‐caspase‐1) and pro‐IL‐1β and pro‐IL‐18. Signal 2 (activation signal; right) is provided by any of numerous damage‐associated molecular patterns (PAMPs) or DAMPs, such as particulates, crystals and eATP. These include K^+^ efflux, Ca^+^ flux and Cl‐ efflux, the decrease of intracellular K^+^ concentration opens the Pannexin‐1, so that microbial molecules enter the cytoplasm and are recognized by LRR to activate the inflammasome. Lysosomal damage can release cathepsin E, mitochondria can products reactive oxygen species (mtROS) and mitochondrial dysfunction releases DNA, these can be recognized by LRR and activate the inflammasome. Activation of the inflammasome activates caspase 1, which cleaves pro‐IL‐1β and pro‐IL‐18 to generate active IL‐1β and IL‐18. Caspase‐1 can cut gasdermin D (GSDMD), and its N‐terminal (GSDMD‐N) can form pores in the cell membrane and induce pyroptosis. Inhibiting Toll‐like receptors (LRs) or tumor necrosis factor receptors (TNFRs) activation, IL‐10 secretion, the binding of miRNA to the NLRP3 and the expression of iNOS gene can negatively regulate the activation of NLRP3. IL, interleukin; NLRP3, LRR, leucine‐rich repeat; NOD‐like receptor pyrin domain‐related protein 3.

The NLRP3 inflammasome is composed of three parts: NLRP3 receptor protein, pro‐caspase‐1, and apoptosis speck‐like protein (ASC). The NLRP3 receptor protein consists of an N‐terminal pyrin domain (PYD), a C‐terminal leucine‐rich repeat (LRR) that can identify PAMPs and DAMPs, and a central NACHT domain (or NOD‐like domain). ASC includes the N‐terminal PYD and C‐terminal caspase activation and recruitment domain (CARD). As an adaptor protein, ASC aggregates into dimers of macromolecules during inflammasome activation, termed ASC‐speck. ASC connects the PYD of receptor protein with the CARD of pro‐caspase‐1 by forming PYD‐PYD domain and CARD‐CARD domain, so as to recruit pro‐caspase‐1 and finally assemble inflammasome (Figure 1). A serine‐threonine kinase named NIMA‐related kinase 7 (Nek7) directly oligomerizes with the NLRP3 receptor protein, it is required for ASC speck formation and activation of caspase‐1.^15^ The ligand binds to LRR, and the NLRP3 inflammasome undergoes a conformational change to hydrolyze pro‐caspase‐1 to generate caspase‐1 that has enzymatic activity. Caspase‐1 cleaves pro‐interleukin(IL)−1β and pro‐IL‐18 to generate active IL‐1β and IL‐18 and promote their secretion^16^ (Figure 1). The production of inflammatory factors can further cause pyroptosis and activation of immune cells such as T cells and NK cells, and release of inflammatory factors such as interferon‐γ (IFN‐γ) and tumor necrosis factor‐α (TNF‐α).^17^, ^18^ These activated cells and inflammatory factors play an important role in the pathogenesis of the disease. In addition, caspase‐1 can cut gasdermin D (GSDMD), and its N‐terminal (GSDMD‐N) can form pores in the cell membrane, resulting in pyroptosis. Active IL‐1 β and IL‐18 are released outside the cells through the pores formed by GSDMD and cause inflammation^17^ (Figure 1).

### 2.2 Activation of the NLRP3 inflammasome

NLRP3 inflammasome activation requires two steps, a first signal and a second signal. The first signal is also called priming signal, which is usually PAMP. Some studies have shown that IL‐1 β and TNF‐ α can also be used as priming signals. They initiate the gene transcription of NLRP3, CASP1, IL‐1β and IL‐18 through the NF‐ κB pathway, and produce more pro‐IL‐1 β and pro‐IL‐18. The second signal is usually DAMP and PAMP, and the activation mechanism mainly includes (1) efflux of K + and influx of Ca^+^ change the cytoplasmic levels of K^+^ and Ca^2+^, extracellular ATP can bind to the ATP‐ligand‐gated channel that called P2X7 on the cell membrane, causing K^+^ efflux, and the decrease of intracellular K^+^ concentration opens the Pannexin‐1, so that microbial molecules can enter the cytoplasm and are recognized by LRR to activate the inflammasome. On the one hand, perforin can cause K^+^ outflow from the cell, on the other hand, it can form pores in the cell membrane so that microorganisms can enter the cell and be recognized by LRR.^19^ In addition to ATP and perforin, alum, silica, calcium pyrophosphate crystals and the membrane attack complex (MAC)^20^ also induce K^+^ efflux. In addition, the influx of extracellular Ca^2+^ into the cytoplasm can also activate the inflammasome.^21^ (2) lysosomal damage, crystals or protein polymers destroy lysosomes and release cathepsin E into the cytoplasm, which is recognized by LRR to activate the inflammasome. (3) ROS production and mitochondrial dysfunction, ROS is a byproduct of normal mitochondrial metabolism and homeostasis,^22^ and mitochondrial damage will release DNA, both of which can be recognized by LRR to activate the NLRP3 inflammasome^18^ (Figure 1). In addition, NADPH oxidase, xanthine oxidase, cytochrome P450, cyclooxygenase, and lipoxygenase, which can also generate ROS to induce NLRP3 activation.^23^

### 2.3 Regulation of inflammatory bodies in NLRP3

The regulation modes of NLRP3 inflammasome mainly include transcriptional regulation, posttranscriptional regulation and posttranslational modification. Transcriptional regulation mainly refers to the transcription initiation regulation of cells dependent on NF‐κB, activation of this pathway enables the NLRP3 receptor protein to reach a functional level before activation, and NF‐κB is regulated by Toll‐like receptors (TLRs) or tumor necrosis factor receptors (TNFRs), which can inhibit the inflammasome by inhibiting TLRs or TNFRs. In addition, type I interferons can promote IL‐10 secretion, and IL‐10 can inhibit the expression of the pro‐IL‐1β, which can inhibit inflammasome activation. posttranscriptional regulation mainly refers to the negative regulation of miRNA. The binding of miR‐233 to the conserved sites in the 3 ‘untranslated region of NLRP3 results in a decrease in the translation of NLRP3 receptor proteins, thereby inhibiting the activation of inflammasome.^24^, ^25^ Posttranslational modification inhibition refers to the expression of iNOS gene induced by type I interferon, reducing the release of ROS by producing NO (Figure1). In addition, studies have shown that the LRR domain of NLRP3 can be ubiquitinated by membrane‐associated RING CH protein VII (MARCH‐7), and phosphorylation of its Ser291 residue can also negatively regulate the activation of NLRP3.^26^

## 3 NLRP3 AND ASTHMA

Asthma is a common allergic respiratory disease, which usually begins in childhood. It is a chronic airway inflammatory disease involving a variety of cells, including airway inflammatory cells (such as eosinophils, mast cells, T lymphocytes, neutrophils) and structural cells (such as smooth muscle cells, airway epithelial cells, etc.) and related cellular components. Pathologically, it is characterized by eosinophilic inflammation and T lymphocyte inflammation involving CD4 aggregation.^27^ This chronic inflammation leads to airway hyperresponsiveness (AHR), which is usually characterized by reversible airflow limitation, and causes recurrent symptoms such as wheezing, shortness of breath, chest tightness or cough, which often occur and aggravate at night and/or early in the morning. Most patients can be relieved by themselves or by treatment. If asthma occurs repeatedly, it can produce a series of changes in airway structure with the prolongation of the course of disease, which is called airway remodeling. Airway remodeling leads to irreversible or partially irreversible airflow limitation and persistent AHR, which reduces sensitivity to inhaled hormone therapy. More and more studies have found that the NLRP3 inflammasome may be involved in the chronic inflammatory process of asthma. Cheng et al.^28^ found that the expression of NLRP3 inflammasome and its downstream products caspase‐1 and IL‐1β increased in the bronchoalveolar lavage fluid (BALF) of ovalbumin (OVA)‐sensitized asthmatic airway inflammation model mice. Wood et al.^29^ found increased expression of IL‐1β and upregulated expression of NLRP3 and NOD‐like domain 1 genes in sputum of 127 patients with asthma.

### 3.1 Activation of the NLRP3 inflammasome in asthma

There is no clear conclusion on how NLRP3 inflammasomes are activated in asthma. In recent years, a large number of studies have explored the activation mechanism of the NLRP3 inflammasome. Kim et al.^10^ developed a mouse model of severe steroid‐resistant allergic airway disease induced by chlamydia, haemophilus and OVA. The results showed that pathogenic microorganism infection such as chlamydia and haemophilus increased the expression of NLRP3, caspase‐1 and IL‐1 β, driving steroid‐resistant neutrophil inflammation and AHR. Rodríguez‐Alcázar et al.^30^ found that Charcot‐Leyden crystals which can be formed after eosinophil degranulation are recognized by the NLRP3 receptor and promote ASC‐involved assembly of the NLRP3 inflammasome and release of IL‐1β, thereby maintaining chronic inflammation after eosinophilic inflammation. Tsai et al.^31^ found that Derf1, a common allergen, not only induces allergy, but also leads to pyroptosis and IL‐1 β secretion in human bronchial epithelial cells (HBECs) through NLRP3‐caspase‐1 pathway. Kim et al.^32^ treated mice with OVA or titanium dioxide nanoparticles (NPs). They found that NPs increased the expression of NLRP3, caspase‐1, IL‐1 β and IL‐18 in mice. Similarly, Ko et al.^33^ treated mice with silica also demonstrated that silica NPs aggravated airway inflammation in asthmatic mice by activating NLRP3 inflammasomes. Wang et al.^34^ explored the effects of follistatin‐like 1 (FSTL1) on NLRP3‐caspase‐1‐IL‐1 β pathway in OVA‐induced mice and macrophages, and found that FSTL1 deficiency can improve OVA‐induced airway mucin MUC5AC production and mucus over secretion, reduce inflammatory cytokines production and inflammatory cell infiltration, and inhibit the production of NLRP3 and IL‐1 β.

### 3.2 Potential inhibitory targets of the NLRP3 inflammasome in asthma

**Figure 2:**
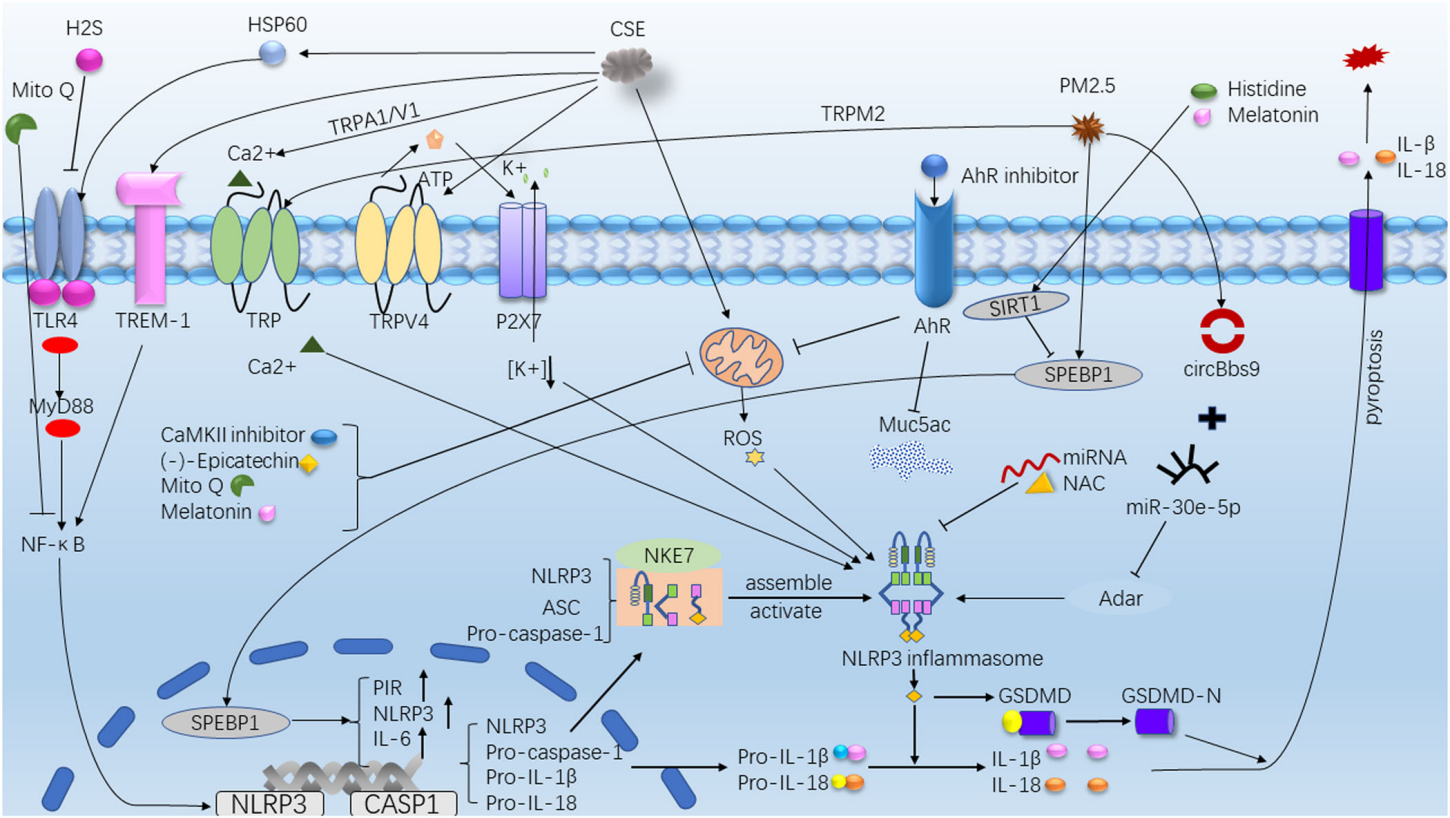
Activation/inhibition Pathway. AhR, aryl hydrocarbon receptor; CaMKII, Ca^2+^/calmodulin‐dependent protein kinase II; CSE, cigarette smoke extract; GSDMD, Gasdermin D; HSP60, heat shock protein 60; Mito Q, mitoquinone; NAC, N‐acetylcysteine; PIR, Pirin; PM, particulate matter; ROS, reactive oxygen species; SIRT1, Sirtuin1; SREBP‐1, sterol regulatory element binding protein‐1; TLR4, Toll‐like receptor 4; TREM‐1, triggering receptor expressed on myeloid cells 1; TRP, transient receptor potential protein.

A large number of experiments have proved that the activation of NLRP3 inflammasome can be inhibited by inhibiting key targets upstream or downstream of the activation pathway, providing new ideas for the treatment of asthma. Wood et al.^29^ conducted a randomized, crossover and acute feeding study on 23 asthmatic adults (*n* = 12 nonobese subjects and *n* = 11 obese subjects). It was found that saturated fatty acid diet significantly increased the 4 h percentage of neutrophils in sputum and the gene expression of TLR4 and NLRP3 in nonobese asthma patients compared with those measured at 0 h. Behavioral interventions to reduce fatty acid exposure, such as losing weight and restricting saturated fat diet, may prevent and treat chronic inflammation in asthma. Studies by Guo et al.^25^ have shown that miR‐22‐3p can be used not only as a negative regulator of NLRP3‐caspase‐1‐IL‐1 β pathway (Figure 2), but also as a protective factor of inflammatory response. ATP and S100 proteins are newly found DAMP in the inflamed respiratory tract. Kim et al.^35^ found that ATP and S100 proteins can promote MUC5AC production, while NLRP3 siRNA expression, NF‐kB pathway inhibitors, NLRP3 inflammasome oligomerization and caspase‐1 inhibitors can almost completely inhibit ATP and S100A12‐mediated MUC5AC production. Lv et al.^36^ found that heme oxygenase‐1 (HO‐1) induced inhibition of Th2 response and reduced apoptosis of HBEC in OVA‐induced eosinophil asthma mouse model. In vitro, HO‐1 induced desensitization of HBEC to OVA‐induced apoptosis, which confirmed the results observed in vivo. It is also proved that HO‐1 binds to the NACHT domain of NLRP3 and RXR α and RXR β subunits, which can inhibit inflammation and maintain cell homeostasis by inhibiting NLRP3‐RXR axis. Suhuang antitussive capsule (Suhuang) belongs to the preparation of Chinese patent medicine. It is mainly composed of Ephedra, Perilla leaves, Pheretima, loquat, Perillae Fructus, Cicadae Periostracum, Peucedani Radix, Arctii Fructus and Schisandrae Chinensis Fructus. In recent years, many studies have found that Suhuang antitussive capsule may have an inhibitory effect on the NLRP3 inflammasome. Qin et al.^37^ found that Suhuang inhibited NF‐κB signal transduction and the activation of NLRP3, it suppressed inflammation by maintaining mitochondrial homeostasis. Qin et al.^38^ sensitized mice with OVA/Al(OH)3, and the experimental group was given different doses of Suhuang. They found that Suhuang can destroy the assembly of NLRP3 inflammasome and reduce the secretion of IL‐1β. Yupingfeng Powder (YPFS) which is a Chinese medicine formula consisting of Astragalus membranaceus, Atractylodes macrocephala, and Saposhnikoviae radix has been found to have therapeutic effect on asthma and with less untoward effects. Liu et al.^39^ proved that YPFS could significantly reduce the mRNA and protein expression of NLRP3, caspase‐1, IL‐1 β and ASC in LPS‐stimulated human leukemia U937 cells. In vivo experiments showed that YPFS treatment could not only alleviate the clinical symptoms of OVA‐sensitized mice, but also reduce inflammatory cell infiltration, mucus secretion and MUC5AC production in BALF of sensitized mice. In addition, some studies have found that estrogen can also inhibit the mRNA and protein expression of NLRP3, caspase‐1 and IL‐1 β. Zhao et al.^40^ induced wild type (WT) and apolipoprotein E (ApoE) deficient mice with OVA to establish a model of allergic airway inflammation. It was found that the activation of NLRP3 inflammasome was further enhanced in OVA sensitized ApoE deficient mice. Gordon et al.^41^ found that ApoE could not induce BALF macrophages to release IL‐1 β below the critical concentration. On the contrary, above the critical concentration of ApoE could activate NLRP3 inflammasome and induce BALF macrophages in patients with asthma to secrete mature IL‐1 β, which further magnified pulmonary inflammatory response. It has been found that OM‐85 which is a bacterial product has a dual inhibitory effect on the production of IL‐1 β. On the one hand, it upregulates the levels of pro‐IL‐1 β and pro‐IL1 α in a MyD88‐dependent manner without activating inflammasome, on the other hand, it inhibits the secretion of IL‐1 β induced by alum which is a common NLRP3 activator.^42^ Leukotriene B4 (LTB4) is the main chemotactic molecule for neutrophil recruitment. Its receptors BLT1 and BLT2 have been thought to contribute to the production of airway inflammation in neutrophil‐dominated asthma. Kwak et al.^43^ established a neutrophil airway inflammation model by attacking mice with house dust mite. It was found that blocking BLT1 or BLT2 could significantly inhibit the activation of NLRP3 inflammasome and the synthesis of IL‐1 β. Similarly, there are studies shown that miR‐223 modulates immune inflammatory responses by inhibiting the NLRP3‐caspase‐1‐IL‐1β pathway.^24^ The Ca^2+^/calmodulin‐dependent protein kinase II (CaMKII) exists in mitochondria and can be activated by ROS to promote eosinophil recruitment and infiltration and AHR. Sebag et al.^44^ established an allergic asthma mouse model. The experimental group was given CaMKII inhibitor. They collected HBEC from OVA‐challenged mice. It was found that mitochondrial CaMKII (mt‐CaMKII) increased the release of mitochondrial ROS (mt‐ROS) and promoted the activation of NLRP3 inflammasome, while mt‐CaMKII inhibitor could reduce the release of ROS (Figure 2), which significantly reduced AHR, eosinophil inflammation and the expression of cytokines induced by Aspergillus fumigatus and OVA. Hu et al.^45^ used cockroach allergen induce asthma mouse model and HBECs and found that inhibition of epithelial aryl hydrocarbon receptors (AhR) could reduce the production of ROS in HBECs, especially mt‐ROS (Figure 2). They point out that the AhR‐ROS‐NLRP3 axis may be a potential target for the treatment of allergic asthma. In addition, studies have shown that CpG oligodeoxynucleotides (CpG‐ODN),^46^ melatonin, abscisic acid (ABA) or foods rich in ABA can also inhibit the activation of NLRP3 inflammasome and delay the progression of airway inflammation.^47^

## 4 NLRP3 AND COPD

COPD is usually caused by smoking and occurs in middle age. It is a preventable and treatable disease with the characteristics of airflow limitation. Airflow limitation is progressive and not completely reversible, which is related to the increased chronic inflammatory response of the airway and lungs to harmful particles or gases. Pathologically, it is mainly characterized by neutrophil inflammation and T lymphocyte and macrophage inflammation involving increased CD8.^48^

### 4.1 Activation of NLRP3 inflammasome in COPD

#### 4.1.1 Cigarette smoke activates NLRP3 inflammasome

Studies have shown that NLRP3 inflammasomes are involved in COPD and acute exacerbation of COPD (AECOPD).^49^, ^50^ Quitting smoking is the most important intervention for COPD patients who smoke. Wang et al.^51^ used cigarette smoke extract (CSE) to induce alveolar epithelial cells (AECs) and HBEC. It was found that CSE increased oxidative stress in mitochondria, induced Ca^2+^ influx, promoted inflammatory gene expression and inhibited antioxidant gene expression. Transient receptor potential protein (TRP) is a calcium permeable cation selective channel. Inhibitors of TRPA1 and TRPV1 and gene knockout reduce oxidative stress, block Ca^2+^ influx and promote antioxidant gene expression. Zhang et al.^52^ used CSE to induce HBEC, they found that CSE through the ROS‐NLRP3‐caspase‐1‐GSDMD pathway induced inflammation and led to pyroptosis (Figure 2). Other studies have found that TRPV4 cationic channels can release ATP and IL‐1 β and induce chronic inflammation in COPD (Figure 2). Rao et al.^53^ found that CSE could induce pyroptosis of mouse airway epithelial cells and up‐regulate the expression of TRPV4. In addition, TRPV4 inhibitor or gene knockout could inhibit pyroptosis mediated by NLRP3‐caspase1‐GSDMD pathway, reduce the expression of IL‐1 β, IL‐8 and IL‐18, alleviate CSE‐induced mitochondrial damage, and increase the expression of anti‐inflammatory genes. It has been found that triggering receptor expressed on myeloid cells 1 (TREM‐1) is an effective magnifier of innate immune inflammatory response. It induces pyroptosis through NLRP3‐caspase‐1‐GSDMD pathway, and promotes lung injury and inflammation in COPD mice^54^ (Figure 2). Moreover, some studies have found that CSE stimulates the expression of heat shock protein 60 (HSP60), activates TLR4‐MyD88‐NF‐κB signal pathway, and then activates NLRP3 inflammasome^55^ (Figure 2). HSP60 has the potential to act as a cellular “danger” signal of immune response.

#### 4.1.2 PM2.5 activates NLRP3 inflammasome

Particulate matter (PM2.5) is one of the most important components of environmental pollutants, but the inflammatory pathway of lung injury caused by PM2.5 is not clear. Tien et al.^56^ treated human lung fibroblast model with PM2.5, and then they found that sterol regulatory element binding protein‐1 (SREBP‐1) is the main downstream regulator of Sirtuin1 (SIRT1). SIRT1 inhibits SREBP‐1 and further downregulates Pirin (PIR) and NLRP3 inflammasome after PM exposure (Figure 2). It is suggested that the SIRT1‐SREBP‐1‐PIR‐NLRP3 axis may be related to the chronic inflammation of COPD, SIRT1 can be used as a protective agent for PM exposure, and PIR can be used as a predictor of cardiopulmonary diseases induced by PM. Li et al.^57^ found that PM 2.5 can regulate the activation of NLRP3 inflammasome through the circBbs9‐miR‐30e5p‐Adar pathway (Figure 2). The study of Wang et al.^58^ also proved the positive role of ROS‐TRPM2‐Ca2 + ‐NLRP3 pathway in PM 2.5‐induced lung injury (Figure 2). The above experiments demonstrate that PM2.5 can activate the NLRP3 inflammasome through multiple pathways, providing a new targeted therapeutic idea for the treatment of PM2.5‐induced lung injury. Neutrophil extracellular traps (NETs) are reticular structures containing DNA and antimicrobial proteins. NETs can discharge neutrophil and contribute to lung inflammation. Wright et al.^59^ collected sputum from healthy controls, asthma and COPD patients and found that NETs existed in the airway of asthma and COPD subjects. Excessive NETs accumulation was related to the activation of innate immune response, leading to chronic airway inflammation. In addition, some studies have pointed out that extracellular heat shock protein 70^60^ (eHsp70) and biofuel smoke^61^ can activate the NLRP3 inflammasome and also participate in the pathogenic process of COPD.

### 4.2 Potential inhibitory targets of the NLRP3 inflammasome in COPD

Melatonin is known to improve sleep and adjust jet lag, Mahalanobish et al.^62^ found that melatonin showed protective effect on COPD both in vitro and in vivo. Melatonin activated intracellular antioxidant thioredoxin‐1 and inhibited the activation of NLRP3 inflammasome that mediated by mitochondrial damage or endoplasmic reticulum stress. Peng et al.^63^ also demonstrated that melatonin attenuates airway inflammation in COPD mice through SIRT1‐dependent inhibition of NLRP3 inflammasome and IL‐1β release (Figure 2). Wang et al.^64^ found that H2S reduces NLRP3 expression and inhibits GSDMD‐mediated pyroptosis by inhibiting TLR4‐NF‐κB‐NLRP3 signal pathway (Figure 2). In addition, H2S can prevent and reverse lung inflammation and emphysema in the alveolar cavity, while preventing but not reversing ozone‐induced airflow limitation and airway remodeling.^65^ Magnesium isoglycyrrhizinate (MgIG) is an anti‐inflammatory glycyrrhizic acid preparation for the treatment of hepatitis. Studies have shown that it can inhibit the activation of inflammasome in NLRP3, suggesting that MgIG may be an alternative to COPD therapy.^66^ Histidine has anti‐inflammatory effect on colitis and other diseases, Tian et al.^67^ found that it can inhibit the activation of inflammasome and control the chronic inflammation of COPD through SIRT1‐NLRP3 pathway. N‐acetylcysteine (NAC) has antioxidant and anti‐inflammatory effects on COPD. Liu et al.^68^ have found that NAC can inhibit the expression of NLRP3 in macrophages (Figure 2) and reduce the production of IFN‐γ in NK cells through clinical trials. Therefore, NAC can effectively inhibit the synthesis and release of IL‐18. (‐)‐Epicatechin (EC) can improve pyroptosis and inflammation induced by CSE through inhibiting ROS‐NLRP3‐caspase‐1‐GSDMD pathway^69^ (Figure 2). Chen et al.^70^ found that mitoquinone (MitoQ) can reduce ROS production and excessive autophagy to maintain mitochondrial function. In addition, MitoQ restores endothelial integrity and inhibits the NF‐κB‐NLRP3 pathway (Figure 2). Isoforskolin (ISOF) is a bioactive component from the plant Coleus forskohlii, native to Yunnan in China. Xiao et al.^71^ established an AECOPD mouse model and found that ISOF treatment could improve lung function and reduce the levels of inflammatory mediators (TNF‐ α, IL‐1 β, IL‐6, IL‐17A, MCP‐1, MIG, IP‐10, and CRP) in lung homogenate, as well as inhibit NF‐ κ B‐NLRP3 inflammatory pathway. Thus, ISOF play a role in preventing and treating AECOPD inflammation. Glucocorticoid is widely used in the treatment of COPD and AECOPD, but its efficacy is low and it is easy to produce hormone insensitivity. 17‐oxo‐DHA is a new anti‐inflammatory drug. It has been proved that 17‐oxo‐DHA combined with hormones can inhibit the activation of NLRP3 inflammasome and the release of mature IL‐1 β.^72^

## 5 EXPECTATION

In addition to asthma and COPD, the NLRP3 inflammasome also involved in the pathogenic process of various diseases. Ji et al.^73^ found that NLRP3 inflammasome in alveolar epithelial cells of patients with idiopathic pulmonary fibrosis and mice with pulmonary fibrosis were activated, which induced lung‐resident mesenchymal stem cells to differentiate into myofibroblasts and promoted the formation of pulmonary fibrosis. Trachalaki et al.^74^ have demonstrated that excessive activation of NLRP3 and AIM2 inflammasome driven by mitochondrial oxidation may be involved in the formation of pulmonary fibrosis. SARS‐CoV‐2 virus has been prevalent all over the world since 2020, and the excessive inflammation caused by SARS‐CoV‐2 infection is related to the severe symptoms of COVID‐19. Studies have shown that the SARS‐CoV‐2 N protein can directly bind to the NLRP3 receptor protein to promote the assembly of the NLRP3 inflammasome and the synthesis and release of IL‐1β.^75^ In addition, Yalcinkaya et al.^76^ pointed out that severe COVID‐19 patients experience cytokine storm and inflammasome activation. SARS‐Cov‐2 E protein may inhibit the host NLRP3 inflammasome response to viral RNA in the early stage of infection, but it will increase NLRP3 inflammasome response in the later stage of infection, and eventually induce severe inflammatory response. The NLRP3 inflammasome is not only involved in the pathogenesis of respiratory diseases, but also has a large number of reports in other systemic diseases, such as atherosclerosis,^77^, ^78^ liver disease,^79^, ^80^ Alzheimer's disease,^81^, ^82^ inflammatory bowel disease^83^, ^84^ and diabetes.^85^, ^86^

So far, there have been reports about NLRP3 inhibitors, MCC950 is the most effective NLRP3 inhibitor. Coll et al.^87^ found that MCC950 directly binds to the NACHT domain of the NLRP3 inflammasome, blocking ATP hydrolysis and inhibiting NLRP3 inflammasome activation. CY‐09 is a selective direct inhibitor of NLRP3. Zhang et al.^88^ have proved that CY‐09 can inhibit pyroptosis mediated by NLRP3 inflammasome. Tranilast is a clinical antiallergic drug. Huang et al.^89^ found that TR can directly bind to the NACHT domain of NLRP3 and inhibit the assembly of NLRP3 inflammasome by blocking NLRP3 oligomerization. Yang et al.^90^ found that β‐carotene binds to the PYD of NLRP3 and selectively inhibits the activation of NLRP3 inflammasome. These studies provide new strategies for the treatment of NLRP3 inflammasome‐related diseases.

However, due to the complex activation mechanism of NLRP3 inflammasome, the inhibition mechanism and precise target of NLRP3 inflammasome have not been fully elucidated. Studies have pointed out that some targeted treatments have significant side effects, such as the suspension of phase II clinical trials of MCC950 in the treatment of rheumatoid arthritis due to hepatotoxicity. Therefore, targeted therapy has potential risks, and the clinical application of targeted therapy needs further research.

## 6 CONCLUSION

In this article, we describe recent findings related to the composition, activation, and regulation of the NLRP3 inflammasome. In addition, we also summarized the latest research results that NLRP3 inflammasomes are involved in the chronic inflammatory process of asthma and COPD. The lung activate the NLRP3 inflammasome by inhaling PAMPs and DAMPs, promoting the release of inflammatory factors such as IL‐1β and IL‐18, and gradually expanding the inflammatory effect and prolonging the duration of inflammation, finally, these would cause chronic inflammation, tissue damage and fibrosis. Currently, a variety of NLRP3 inflammasome inhibitors have been reported, including direct inhibitors or indirect inhibitors of inflammasome, inhibitors can block NLRP3 by binding to key targets upstream or downstream of its activation pathway. However, clinical safety needs to be further explored. With the continuous understanding of the mechanism of activation of NLRP3 inflammasome, accurate drug therapy is the trend in the treatment of inflammatory diseases, so the direct specific inhibitors of NLRP3 inflammasome is worthy of further research and trial.

## AUTHOR CONTRIBUTIONS

Yaxin Wu and Ke Wang designed the research study. Yaxin Wu, Xin Di, Min Zhao, and Haoran Li performed the research. Min Zhao, Haoran Li, and Li Bai contributed critical review of the intellectual content of the article. Yaxin Wu, Xin Di, and Ke Wang contributed to the writing of the manuscript. All authors read and approved the final manuscript.

## CONFLICT OF INTEREST

The authors declare no conflict of interest.

## Data Availability

Data sharing is not applicable to this article as no new data were created or analyzed in this study.
